# Toward applications of near-field radiative heat transfer with micro-hotplates

**DOI:** 10.1038/s41598-021-93695-7

**Published:** 2021-07-12

**Authors:** Olivier Marconot, Alexandre Juneau-Fecteau, Luc G. Fréchette

**Affiliations:** 1 Institut Interdisciplinaire d’Innovation Technologique (3IT), Université de Sherbrooke, Sherbrooke, QC J1K 0A5 Canada; 2 Laboratoire Nanotechnologies Nanosystèmes (LN2) - CNRS UMI-3463, Université de Sherbrooke, Sherbrooke, QC J1K 0A5 Canada

**Keywords:** Nanophotonics and plasmonics, Nanosensors, NEMS, Polaritons, Sub-wavelength optics

## Abstract

Bringing bodies close together at sub-micron distances can drastically enhance radiative heat transfer, leading to heat fluxes greater than the blackbody limit set by Stefan–Boltzmann law. This effect, known as near-field radiative heat transfer (NFRHT), has wide implications for thermal management in microsystems, as well as technological applications such as direct heat to electricity conversion in thermophotovoltaic cells. Here, we demonstrate NFRHT from microfabricated hotplates made by surface micromachining of $\hbox {SiO}_2$/$\hbox {SiN}$ thin films deposited on a sacrificial amorphous Si layer. The sacrificial layer is dry etched to form wide membranes (${100}\,\upmu \hbox {m} \times {100}\,\upmu \hbox {m}$) separated from the substrate by nanometric distances. Nickel traces allow both resistive heating and temperature measurement on the micro-hotplates. We report on two samples with measured gaps of ${610}\,\hbox {nm}$ and ${280}\,\hbox {nm}$. The membranes can be heated up to ${250}\,^{\circ }\hbox {C}$ under vacuum with no mechanical damage. At ${120}\,^{\circ }\hbox {C}$ we observed a 6.4-fold enhancement of radiative heat transfer compared to far-field emission for the smallest gap and a 3.5-fold enhancement for the larger gap. Furthermore, the measured transmitted power exhibits an exponential dependence with respect to gap size, a clear signature of NFRHT. Calculations of photon transmission probabilities indicate that the observed increase in heat transfer can be attributed to near-field coupling by surface phonon-polaritons supported by the $\hbox {SiO}_2$ films. The fabrication process presented here, relying solely on well-established surface micromachining technology, is a key step toward integration of NFRHT in industrial applications.

## Introduction

Near-field radiative heat transfer (NFRHT) occurs between objects separated by a distance smaller than the wavelength of thermal photons. Some materials (as $\hbox {SiO}_2$ or $\hbox {SiC}$^1^) support Surface Phonon Polaritons (SPhPs), which are electromagnetic surface waves resulting from coupling between light and crystal vibrations. These waves are strong carriers of electromagnetic energy and are confined on the interfaces. By approaching two materials which support similar SPhPs, a coupling of surface waves could drastically enhance the electromagnetic energy transfer, leading to NFRHT^2^. In this regime, radiative coupling from evanescent waves emitted by both bodies enhances transmitted power by up to a factor 100 at nanometer-scale gaps (${50}\,\hbox {nm}$) compared to the blackbody limit of conventional far-field heat transfer^3,4^. Several studies have shown that NFRHT can be harnessed for applications such as thermophotovoltaic energy conversion^5–9^, enhanced radiative cooling^10^ and thermal rectification^11–15^. Keeping two objects with a large temperature difference separated by only a few hundred nanometers remains a challenging task. Successful approaches include precise piezoelectric actuators with feedback control loops to keep both objects apart^16–18^ or arrays of spacers between the surfaces^19–21^. The first option is difficult to scale to commercial applications as it requires bulky and expensive equipment, whereas the second implies high heat conduction losses through the spacers and mechanical stress from thermal expansion. In both cases, gap inhomogeneities due to surface curvatures is an issue as well. Microfabrication processes used to manufacture microelectromechanical systems (MEMS) can mitigate many of the previously mentioned problems by allowing high precision suspended structures with sub-microns gaps, compatible with industrial production. MEMS technology has been successfully used to observe NFRHT across submicron distances between suspended beams^22,23^ or membranes^7,24,25^. However, to increase the heat transfer while minimizing conduction losses, large areas combined with good thermal insulation are required. One such type of thermal MEMS is the micro-hotplate, a thin film membrane suspended by long thin legs with a resistive heater on top. The high thermal resistance of the legs provides good insulation from thermal losses to the environment through conduction, allowing the membrane to reach high temperatures for low heating powers. Due to this insulation and the high surface to mass ratio, the thermal response of the micro-hotplate is very sensitive to gas conduction and radiative heat transfer. This property is exploited in highly accurate Pirani pressure gauges^26,27^ and micro-bolometers for uncooled infrared imagery^28^. Moreover, in-plane integration allows fabrication of large membrane arrays thanks to surface micromachining techniques. Although this geometry is widespread, few experimental studies of NFRHT from micro-hotplates have been realized. Feng et al. demonstrated that the presence of a second membrane ${1}\,\upmu \hbox {m}$ above a micro-hotplate affects the heating rate^29^. They attribute this effect to NFRHT between the suspended structures. However, they did not characterize radiative heat exchange to the substrate from the bottom of the membrane, which is expected to dominate heat losses. Furthermore, near-field heat fluxes decrease exponentially with distance and are expected to be weak at ${1}\,\upmu \hbox {m}$. Thus, for thermal rectification or thermophotovoltaic applications, the required separation distance should be below ${500}\,\hbox {nm}$ to insure good device performance. Song et al.^24,25^ demonstrated NFRHT enhancement between polar dielectric thin films on piezoelectric actuators with an emitter to receiver separation of ${20}\,\hbox {nm}$. However, they had to keep a temperature rise below ${10}\,\hbox {K}$ to prevent thermal buckling.

In this article, we present clear evidence of strong NFRHT both across sub-micron gaps and with more than ${100}\,\hbox {K}$ temperature differences between micro-hotplates and the bulk substrate. We characterize the thermal properties of the device, which we name the NF-hotplate, and directly measure the gap depth over the span of the membrane with a laser optical interferometer. This data draws an accurate picture of gap inhomogeneities according to realistic design parameters for industrial fabrication. We confirm the enhancement of radiative heat transfer due to near-field effect through an exponential increase in radiative heat flux as the distance between the membrane and the substrate decreases, consistent with electrodynamic simulations using the measured gap distributions. Our study points to micro-hotplates as a viable option to leverage NFRHT in practical applications.

**Figure 1:**
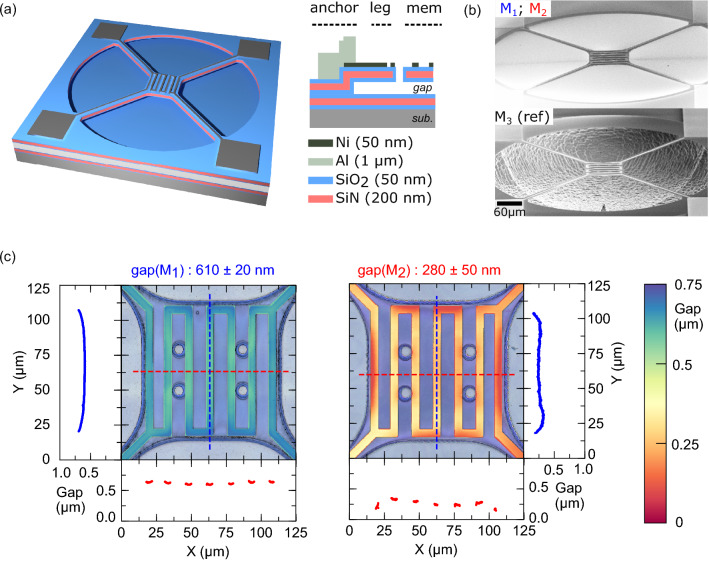
(**a**) 3D and cross section schematic view of the suspended membranes. (**b**) SEM image of a sample with a sub-micron gap such as M1 or M2, and the reference membrane with the substrate etched (M3). (**c**) Superposition of optical microscope images and laser interferometry gap measurements for samples M1 and M2. Gap values measured on the Ni heater are shown by the colormap and also plotted along the horizontal (red) and vertical (blue) dash lines on both images.

## Results

### Fabrication and gap characterization

The samples in this study are square membranes, ${100}\,\upmu \hbox {m}$ × ${100}\,\upmu \hbox {m}$ wide and ${300}\,\hbox {nm}$ thick, with four supporting arms at the corners, as shown schematically on Fig. 1a. These structures were fabricated by plasma enhanced chemical vapor deposition (PECVD) and etching of thin films on $\hbox {Si}$ wafers^30^. The detailed process flow is provided in Supplementary Information S1. The cross-section in Fig. 1a shows that a membrane consists of three layers: ${50}\,\hbox {nm}$ of $\hbox {SiO}_2$, ${200}\,\hbox {nm}$ of $\hbox {SiN}$ and ${50}\,\hbox {nm}$ of $\hbox {SiO}_2$. Such a symmetric stack prevents buckling due to unbalanced out-of-plane mechanical moments. The ${300}\,\hbox {nm}$ total stack thickness is a tradeoff to maximize radiation/conduction ratio and to have a significant radiative heat flux to measure (see Supplementary Information S2). The $\hbox {SiN}$ membrane tensile core ensures mechanical stability, while the $\hbox {SiO}_2$ shell purpose is to sustain SPhPs surface waves for radiative heat transfer. To provide coupling of the evanescent fields with the substrate, it is coated with the same three layers. The deposited $\hbox {SiN}$ is under a tensile stress of approximately ${400}\,\hbox {MPa}$ according to the measured deformation of a $\hbox {Si}$ wafer coated with a single $\hbox {SiN}$ film. The deposited $\hbox {SiO}_2$, on the other hand, is under ${280}\,\hbox {MPa}$ compressive stress. Thus, the trilayer membrane is expected to be under a net ${170}\,\hbox {MPa}$ tensile stress. Such a high tension is beneficial to achieve a flat suspended structure. A PECVD amorphous silicon (a–Si) film acts as the sacrificial layer on which the $\hbox {SiO}_2$/$\hbox {SiN}$/$\hbox {SiO}_2$ membrane trilayer is deposited. $\hbox {SiN}$ core was patterned using $\hbox {SF}_6$ etching prior to depositing the second $\hbox {SiO}_2$ layer. By this way, $\hbox {SiN}$ walls are protected with $\hbox {SiO}_2$ and no etching of the $\hbox {SiN}$ core was observed during membrane release. The square membrane of the NF-hotplate and its supporting arms are defined by photolithography followed by a $\hbox {SF}_6$ plasma etch. Prior to release, a Ni resistive heater is fabricated on the hotplate by lift-off. This heater is connected to aluminum pads by four traces on the arms to provide high quality ohmic contacts. Finally, the a–Si layer is etched by an isotropic $\hbox {SF}_6$ ion coupled plasma to release the membrane. To facilitate the release, the etching pressure is maintained at ${7}\,\hbox {Pa}$ with no platen power to maximize the ratio of chemical to mechanical etch rates^31^. At ${600}\,\hbox {W}$ of coil power, the a–Si etch rate is about ${3}\,\upmu \hbox {m}$/min with a–Si/$\hbox {SiO}_2$ selectivity of more than 100:1. We remark that this process does not consume expensive $\hbox {XeF}_2$, contrary to other nanogap fabrication techniques involving silicon dry etching. A scanning electron microscope (SEM) image of a typical NF-hotplate is shown on Fig. 1b. We studied two devices, M1 and M2, with designed gaps of ${250}\,\hbox {nm}$ and ${750}\,\hbox {nm}$ respectively, as well as another sample, M3, used as a far-field reference. As shown on Fig. 1b, we performed a deep etch of the substrate under the membrane of M3 to create a large cavity preventing near-field transfer.

The gap between the released membrane and the substrate is subject to variations because of unequal mechanical stress distribution. Since a precise value of this parameter must be known to accurately model NFRHT, we used a VK Keyence 3D laser confocal scanning microscope to map the gap across the fabricated membrane, as shown on Fig. 1c. This method was already use to characterize membrane planarity for in-plane far field heat transfer^32^ but was never used for gap characterization for NFRHT measurement. The gap can only be measured on the Ni heater because it is the only material reflecting the laser beam on the membrane. The gap is computed by subtracting the membrane thickness from the heater height measured relative to the substrate. We studied the gap distribution within a region of interest (ROI) which excludes the edge of the membrane. Pixel heights distribution in the ROI were fitted with a normal distribution to extract the mean gap value ($\mu$) and variance ($\sigma$) of the gap distribution. We determine the following gap distribution for M1 and M2 devices : $610\pm {20} \,\hbox {nm}$ and $280\pm {50} \,\hbox {nm}$ respectively, for initial a-Si sacrificial layer thicknesses of ${750} \,\hbox {nm}$ and ${250} \,\hbox {nm}$.

**Figure 2:**
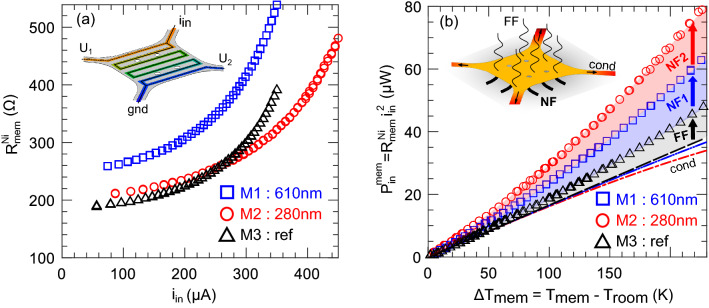
(**a**) Measured heater resistance as a function current for samples with nanometric gaps M1 (blue), M2 (red) and the reference sample M3 (black). Inset: electric potential distribution and the four-point probe measurement. (**b**) Measured input heating power as a function of the temperature increase in the membrane is shown by markers for samples M1 (blue), M2 (red) and M3 (black). Calculated thermal conduction through the legs is shown by the dashed lines. Increases in dissipated power from far-field (FF) and near-field (NF) thermal radiation are indicated by arrows. Inset: temperature distribution and heat fluxes schematic.

### Thermal characterization

We measured heat transfer from the NF-hotplates with the four-point probe method, each electrical probe being a wire on a supporting arm of the membrane. We wire-bonded the NF-hotplates on custom Al core printed circuit boards (PCBs). This assembly was introduced in a vacuum chamber evacuated at $10^{-2}$ Pa to limit heat losses through the air. A DC electric current ($i_{in}$) flows through the nickel and the membrane heats up. By locally measuring the voltage in the membrane center, thanks to probes (inset in Fig. 2a), we deduced the heater electrical characteristic $R_{Ni}^{mem}=f(i_{in})$ (Fig. 2a). Another approach, based on frequency thermal analysis (3 omega), offers highly accurate measurements for low temperature variations^33^. However, in our case the AC technique is not applicable because the devices reach high temperatures (${250}\,^{\circ }\hbox {C}$). Multiple harmonics of the output voltage prevent a direct link of the $3\omega$ temperature rise to the $2\omega$ output voltage. Therefore, we chose to use a steady-sate 4-point probe measurement of temperature to determine the thermal losses. We estimated the accuracy on the probe voltage to be ${200} \,\upmu \hbox {V}$ leading to a ${4} \,\hbox {K}$ error at low power and less than ${1} \,\hbox {K}$ for high power. To stabilize the nickel thermistor for high temperatures, current sweeps with a high maximum value were applied to heat up the membrane until the electrical characteristic was stable. Ni’s Temperature Coefficient of Resistance (TCR) was measured after annealing and permits to deduce the thermal characteristic for the three tested membranes (see “From electrical characteristic to thermal characteristic” section in “Methods” section). Input thermal power is the Joule heating of the Nickel thermistor $P_{in}^{mem}=R_{mem}^{Ni} i_{in}^2$. Temperature elevation of the membrane $\Delta T_{mem}=T_{mem}-T_{room}$ is deduced from the Nickel TCR and the zero-power electrical resistance. The thermal characteristic $P_{in}^{mem}=f(\Delta T_{mem})$) is plotted on Fig. 2b. The input power is dissipated by conduction through the legs and by radiation with the substrate or with the environment (depicted on FEM model inset in Fig. 2b). For the far field reference, radiation is assumed much smaller than conduction for low temperature elevation. We fitted the equivalent thermal conductivity of the legs $\lambda _{leg}^{eq}= 5.2 \, W m^{-1} K^{-1}$ (detailed in “Thermal balance and conduction losses through the legs” section in “Methods” section). By applying a thermal balance on legs and the membrane, the conduction loss for each membrane is found and plotted with dashed lines on Fig. 2b. Conduction losses through the legs decrease when the cross section area is reduced or the length is increased. We fabricated membranes with ${340}\,\upmu \hbox {m}$ legs but deformation was noticeable and membranes move away from the substrate. In addition, conduction losses can be significantly decreased by increasing the electrical resistance, since more Joule heating will occur along the legs. This has for effect of increasing the leg temperature and therefore reducing the tendency for heat to conduct away from the membrane. We observe that for the studied membranes as the conduction loss deviates from a linear trend with temperature. By subtracting membrane input power and modelled conduction accounting for heat generation along the legs, we determine the radiative heat transfer for the three membranes. The membrane over a cavity (M3) only exhibits far field radiative heat transfer, so it acts as a reference. We notice that radiation losses increase as the gap decreases due to near field thermal radiation (arrows in Fig. 2b), proving existence of a strong thermal coupling at sub-micron separation distances. In addition, the membranes show good mechanical stability up to ${270}\,^{\circ }\hbox {C}$, with a monotonic and a reversible behavior.

### Theoretical modelling

We present in this section a theoretical model of heat transfer of the fabricated NF-hotplates for comparison with experimental data, the net heat flux radiated by the membrane is the sum of the thermal energy exchanged with the substrate across a sub-micron distance and the environment, which is considered as an ideal blackbody located far above the membrane. Thermal radiation within a nanoscale gap between flat surfaces is affected by wave interference due to multiple reflections as well as photon tunneling from evanescent coupling. Both effects are taken into account by the fluctuational electrodynamic formalism first introduced by Rytov^3^ and further developed by several authors^4,34,35^. According to this theory, the radiative heat exchange between two objects at respective temperatures $T_1$ and $T_2$ is given by a Landauer-type formula^36^. The transmission probabilities of a complex multilayer structure such as the NF-hotplate can be decomposed as a product of scattering matrices describing photon propagation through each layer. From the scattering matrices calculations, detailed in Supplementary Information, we obtain photons transmission probabilities, noted ${\hat{T}}$, with values between 0 and 1. We assume that the environment is a blackbody at a temperature $T_{room}$, and that the substrate is at the same temperature. Due to the input heating power, the membrane is at a higher temperature $T_{mem}$. Thus, the difference in Planck thermal distributions $\Delta n(\omega )$, between the hot membrane and its cold environment is:

1 $$\begin{aligned} \Delta n(\omega ) = \frac{1}{e^{\hbar \omega /k_bT_s}-1} - \frac{1}{e^{\hbar \omega /k_bT_m}-1} \end{aligned},$$

with $k_b$ the Boltzmann constant, $\hbar$ the reduced Planck constant and $\omega$ is the photon frequency. It is important to note that the membrane is semi-transparent, meaning that the membrane not only absorbs or emits photons but can also transmit them. For instance, one photon could be emitted directly by the membrane and absorbed or reflected by the substrate. Reflected photons are then absorbed, transmitted to the environment or reflected back by the membrane. The probability of photon $(\omega ,k_{||})$ transmission from the membrane to the substrate/environment depends of:

- The probability ${\hat{T}}_s$ (or ${\hat{T}}_e$) of photon emission from the membrane; environment assembly (or membrane, substrate assembly) multiplied by the probability of photon absorption on the substrate (or environment).
- Transmission of photons from environment to substrate and vice versa through the membrane according to probabilities. These probabilities are associated to ${\hat{T}}_m^+$ (or ${\hat{T}}_m^-$) coefficient.

The overall spectral flux density $q(\omega )$ radiated away by the membrane is:

2 $$\begin{aligned} q(\omega ) =&\sum _{\text {TE},\text {TM}} \frac{\hbar \omega }{4\pi ^2}\Delta n(\omega ) \int _0^\infty k_{||}\left( {\hat{T}}_e + {\hat{T}}_s - {\hat{T}}_m^+ - {\hat{T}}_m^-\right) \text {d}k_{||} \end{aligned},$$

where the integration is performed over $k_{||}$, the wave vector component parallel to the surface, and the contributions from both polarization states TE and TM are summed. As the membrane stack is symmetric and as the environment is considered as a blackbody, we have in this work ${\hat{T}}_m^+= {\hat{T}}_m^-$^37^. As the distance between the substrate and the membrane is less than ${1} \,\upmu \hbox {m}$, evanescent waves occur only for the ${\hat{T}}_s$ calculation.

**Figure 3:**
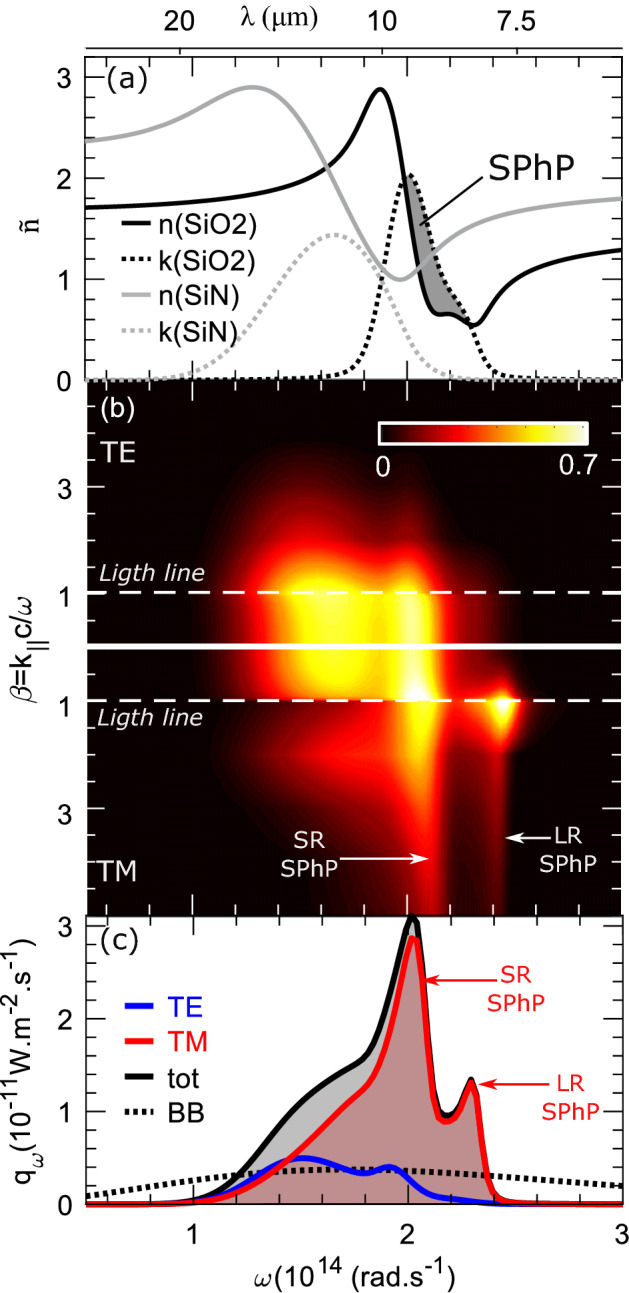
(**a**) Measured complex refractive (full line: real part Re(n), dashed line: imaginary part Im(n)) indexes of $\hbox {SiN}$ (grey) and $\hbox {SiO}_2$ (black) with a IR-Vase ellipsometer. The region supporting SPhPs, where Im(n)> Re(n), is highlighted for $\hbox {SiO}_2$. (**b**) Total transmission probability of a photon emitted by the membrane and transmitted to the substrate (with a gap of ${280} \,\hbox {nm}$) or to the environment. Probabilities are calculated in the $(\omega , k_{||})$ space as thermal photons could be emitted in all directions. For more clarity, $\beta$ is the normalization of $k_{||}$. For $\beta <1$, $k_{||}<\omega /c$ and the wavenumber is a pure real number so the thermal photon correspond to a propagative wave, for $\beta >1$, thermal photon is evanescent and both long range (LR) and short range (SR) SPhPs of $\hbox {SiO}_2$ are visible in TM polarization. (**c**) Spectral radiative heat transfer for TE (blue), TM (red) and total (black) after integration of transmission probabilities with $T_{env}=293 \text{K}$ and $T_{mem}=393 \text{K}$. Integration over $\omega$ gives the net heat flux. For comparison, the spectral density of a perfect black body is plotted (dash line).

To calculate each probability in the $(\omega ,k_{||})$ space and to ensure a good stability for evanescent waves, we used the scattering matrices formalism to calculate transmission and reflection complex coefficients $(\tau ,\rho )$ for the membrane and for the two-body assemblies. The detailed theory for a general geometry with three body at three different temperature is shown in Supplementary Information. We used the measured refractive index of $\hbox {SiO}_2$ and $\hbox {SiN}$ to lead the theoretical calculations (Fig. 3—ellipsometry measurements in “Complex refractive index of SiO_2_ and SiN in mid infrared” section in “Methods” section). Figure 3b represents the overall transmission probability ${\hat{T}}_{tot}={\hat{T}}_{s}+{\hat{T}}_{e}-2{\hat{T}}_{m}$ from the membrane to the environment for a ${280} \,\hbox {nm}$ gap. $\beta =k_{||}c/\omega$ is the dimensionless value of the wave vector parallel component. The total transmission probability matrix has 0 as lower limit and 0.7 for the upper limit proving the good code stability. Representation of ${\hat{T}}_{tot}$ probability is separated in two domains:

- For $\beta <1$, the wave vector is a pure real number corresponding to propagative waves. We observe a strong transmission probability for waves near the absorption region of silicon nitride and silicon oxide (around 0.7 near ${10} \,\upmu \hbox {m}$). For comparison, a perfect blackbody has a transmission probability of 1 for all propagative waves.
- For $\beta >1$, the wave vector is a pure imaginary number corresponding to evanescent waves as frustrated modes or surface phonon-polaritons (SPhPs). Frustrated modes are closed to the light line and exist for both polarizations regardless of the material. The existence of these evanescent waves are not take into account for the blackbody theory.

SPhPs exist in the frequency range where the complex refractive index of silicon oxide satisfies the condition $k > n$, meaning that the real part of the permittivity is negative (highlighted on Fig. 3a). For TM polarization, SPhPs extend far away from the light line. These surface waves have a major impact on the total radiated heat flux since the transmission probability remains nonzero for large $\beta$ (highly confined waves but high energy carrier). The heat flux spectral density (Fig. 3c) is the integral over $k_{||}$ of the overall transmission probability at a particular frequency and polarization (TM: red; TE: blue; total: black). We attribute the double peak in the TM contribution to a splitting of the SPhPs dispersion relation into long-range (LR) and short-range (SR) branches due to coupling between surface waves at both interfaces of each $\hbox {SiO}_2$ film^38^. This effect increases with decreasing $\hbox {SiO}_2$ thickness, as shown in Supplementary Information S2. As a reference, we also plot in Fig. 3c the radiated heat flux from a perfect blackbody according to Planck’s law (dash line). Because of SPhPs, $q(\omega )$ greatly exceeds the blackbody limit from $\omega =1.2\times 10^{14}$ to $2.4\times 10^{14}$ rad/s. The overall heat flux lost by the membrane to the substrate and environment is calculated by integrating over frequency.

**Figure 4:**
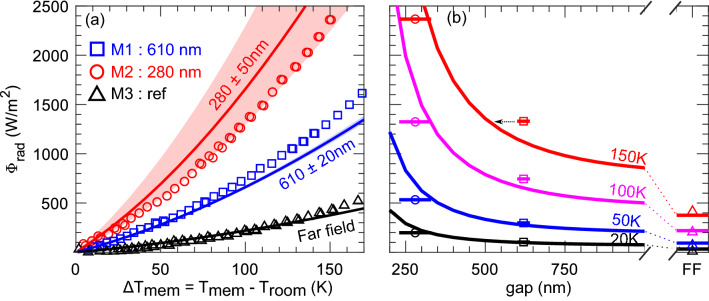
(**a**) Radiative heat flux VS membrane temperature rise. Points correspond to experimental data (M1: blue, M2: red, M3: black). Lines correspond to theoretical calculations for the mean gap and fill correspond to the uncertainty on the gap. (**b**) Radiative heat flux VS gap with the substrate for different temperature rises. Lines are the theoretical calculations and points are the experimental data ($\square$ M1 ; $\circ$ M2 ; $\triangle$ : M3). Arrows represent an eventual thermal buckling leading to a gap variation for high temperatures.

## Discussion

Integration of the theoretical heat flux spectral density over $\omega$ gives us a direct estimation of the net heat flux from the membrane to the environment or the substrate, uncertainty due to the gap distribution ($\mu \pm \sigma$) is represented with the color fills on Fig. 4a. Since the radiation flux increases exponentially as the gap decreases, the gap uncertainty has a strong impact for the smaller gap (${280} \,\hbox {nm}$) compared to the other tested gap. The experimental radiation flux is deduced from the difference between the input power and conduction losses and then normalized to the membrane surface (points on Fig. 4a). Direct comparison of experimental data and modelling confirm the observation of the radiative flux increase due to the near-field radiation. For example, at $\Delta T_{mem}=100 \text{K}$, the membrane with a gap around ${610} \,\hbox {nm}$ (M1) and ${280} \,\hbox {nm}$ (M2) have respectively a net measured radiative heat flux of ${720} \,\hbox {Wm}^{-2}$ (theory : ${770} \,\hbox {Wm}^{-2}$) and ${1260} \,\hbox {Wm}^{-2}$ (theory : ${1680} \,\hbox {Wm}^{-2}$). These values represent respectively a 3.5- and 6.4-fold enhancement compared to the far field reference (M3). For $\Delta T_{mem}=20\,\text{K}$, the measured experimental radiation is in good agreement with the theoretical one (Fig. 4b). Above $\Delta T_{mem}=50\,\text{K}$ the experimental behavior of the two NF hotplates seems to deviate progressively from the theoretical value. We believe that thermal deformation during heating coupled with the high sensitivity of radiative exchange for small gaps is the main reason to the disparity between experimental and theoretical values at higher temperatures. As shown by St-Gelais et al^23^, high tensile stress is required to avoid thermal buckling for large temperatures. For a given $\Delta T_{mem}$, the average stress in the structure is given by:

3 $$\begin{aligned} \sigma _{avg}(\Delta T_{mem})=\frac{t_{SiN}[\sigma _{SiN}-\alpha _{SiN}E_{SiN}\Delta T_{mem}]+t_{SiO_2}[\sigma _{SiO_2}-\alpha _{SiO_2}E_{SiO_2}\Delta T_{mem}]+t_{Ni}[-\alpha _{Ni}E_{Ni}\Delta T_{mem}]}{t_{SiN}+t_{SiO_2}+t_{Ni}} \end{aligned},$$

with $\sigma _i$ the prestress, $t_i$ the thickness of layers, $\alpha _i$ the thermal expansion coefficient ($\alpha _{SiN}=1.6\,\text{ppm/K}$, $\alpha _{SiO_2}=0.5\,\text{ppm/K}$ and $\alpha _{Ni}=13\,\text{ppm/K}$)^39^ and $E_i$ the Young modulus ($E_{SiN}=300 GPa$, $E_{SiO_2}=87 \text{GPa}$^39^ and $E_{Ni}=205 \text{GPa}$^40^). We estimated that the structure falls in compressive stress, so in possible thermal buckling, around $\Delta T_{mem}=200\, \text{K}$. According to Fig. 4b, for $\Delta T_{mem}=150\, \text{K}$, the deviation of experiment from theory correspond to a membrane displacement of ${50}\,\hbox {nm}$ and ${80}\,\hbox {nm}$, respectively for ${280}\,\hbox {nm}$ (M2) and ${610}\,\hbox {nm}$ (M1) initial gaps. Moreover, contribution of the non-ideal geometry (holes and nickel serpentine heater) could also explain the slight deviation of experimental results from theory^41^.

Overall, our study offers a step forward to achieve thermal management with static MEMS using NFRHT. The objective is to build thermal equivalents of standard microelectronic components such as transistors, diodes and memories^14,42^. Instead of using voltages and electric currents, these thermotronic devices perform calculations and information treatment with temperatures and thermal heat fluxes. These new concepts are relevant for thermal imaging using uncooled bolometer technology; the integration of thermal logic gates directly on the bolometers could avoid the use of CMOS readout circuits for in-situ and real-time image processing. In addition, the thermal circuit could perform autonomous calculations by directly using radiation from the environment as a primary energy source instead of the electrical energy used in microelectronics^37^. To build a thermal transistor with MEMS technology, the first requirement is to have a three-body configuration with a semi-transparent membrane as we have shown in this work, prior to integrate a metal insulator transition material (MIT) such as vanadium dioxide.

## Conclusion

We have demonstrated in this work evidence of near field thermal radiation on static semi-transparent membranes. Use of a scalable microfabrication process permits to avoid the use of micro actuators to bring the membrane close enough to the substrate to observe a thermal near field regime. These new devices could facilitate the integration of near field thermal radiation in real application, as we removed the complexity of micro actuator control. The process also allows large membranes to be formed with sub-micron gaps for increased heat transfer. In comparison of propagative radiation (far field reference), we observed a 6.4 times enhancement for a gap around ${300} \,\hbox {nm}$ and 3.5 times higher for larger gap (around ${600} \,\hbox {nm}$) which is in agreement with theoretical calculations based on transmission probability theory for a three body configuration. Membranes reach a maximum temperature of ${270}\,^{\circ }\hbox {C}$ and a good reversibility after thermal cycling. In addition, the membranes are fabricated in the substrate plane, which allows both MIT material integration as well as stack of different membranes coupled in near field to achieve multi body coupling. This study is a step forward to thermal logical gates for autonomous calculation using radiative heat flux, a class of thermotronics.

## Methods

### From electrical characteristic to thermal characteristic

**Figure 5:**
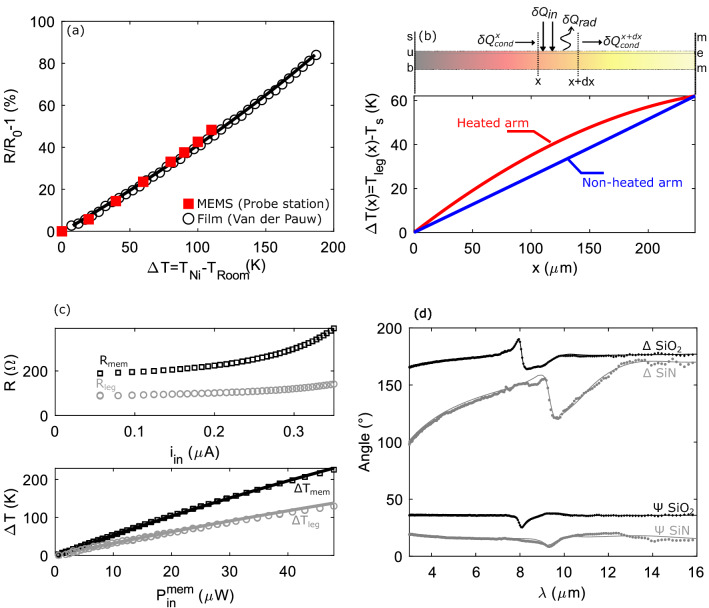
(**a**) TCR of the nickel resistor with two measurement methods: directly on MEMS using a heating four-probe station ($\square$) and on an annealed blanket thin film using a Van der Pauw configuration ($\circ$). (**b**) Thermal balance in legs with the temperature rise profile for heated (red) and unheated (blue) legs for modelling of conduction losses from the membrane through legs. (**c**) Far field membrane resistance and heated leg resistance function of applied current. Experimental thermal characteristic for legs (average temperature, grey circles) and for the membrane (black square). Corresponding thermal model with $\lambda _{leg}^{eq}= 5.2 \, W m^{-1} K^{-1}$. (**d**) Ellipsometry ($\Delta$ and $\Psi$) angles of $\hbox {SiO}_2$ (black) and $\hbox {SiN}$ (grey) respectively with a 40$^{\circ }$ and 65$^{\circ }$ incidence angle. Points are the experimental data and lines represent the fit using Brendel oscillators.

Nickel TCR was measured directly on the MEMS devices after annealing using a heating stage four-point probe station (Fig. 5a). It was also verified on a blanket Nickel thin film annealed at ${350}\,^{\circ }\hbox {C}$ under nitrogen using a Van Der Pauw setup. TCR is not linear above ${100}\,^{\circ }\hbox {C}$ and the resistivity follows the empirical law:

4 $$\begin{aligned} R_{Ni}(\Delta T)=R_{Ni}(293 \text{K})[1+0.0036\Delta T +4.58 \times 10^{-6} \times\Delta T^2] \end{aligned}.$$

Nominal values of Nickel resistor $R_{Ni} (293 \text{K})$ are determined by curve fitting of the electrical characteristic (Fig. 2a). Temperature rise of the membrane $\Delta T_{mem}$ is then found by determining the positive polynomial root of Eq. (3). Membrane input heating power is determined by Joule heating:

5 $$\begin{aligned} P_{in}^{mem}=R_{mem}^{Ni}(\Delta T)\times i_{in}^2 \end{aligned}.$$

### Thermal balance and conduction losses through the legs

The input heat flux, $P_{in}$, is dissipated by conduction, $P_{cond}$, through legs or by radiation, $P_{rad}$, to environment or substrate. These two dissipation heat fluxes depend on the temperature rise $\Delta T_{mem}$ of the membrane, and the thermal balance on the membrane is:

6 $$\begin{aligned} P_{in}(\Delta T_{mem})=P_{cond}(\Delta T_{mem})+P_{rad}(\Delta T_{mem}) \end{aligned}.$$

To determine radiation losses, conduction losses should be perfectly known. As two legs are used as current probes and nickel presents an electrical resistance $R_{leg}^{Ni}$, Joule power is dissipated in these legs. In addition, radiation losses exist along the four legs. These two thermal fluxes could change the temperature profile in the legs, and therefore affect the conduction losses from the membrane. Thermal balance was done on a slice of width *dx* along the leg to formulate the differential equation for the temperature rise in the leg $\Delta T_{leg}$ (Fig. 5b). As boundary conditions, we supposed that the anchor ($x=0$) is at room temperature and the membrane/leg frontier ($x=L_{leg}$) is at the temperature of the membrane. The temperature rise $\Delta T_{leg}$ follows the differential equation system:

7 $$ODE:\frac{{d\Delta T_{{leg}}^{2} }}{{d^{2} x}} - \beta \Delta T_{{leg}} = - \alpha \:{\text{with}}\:\alpha = \frac{{R_{{leg}}^{{Ni}} i_{{in}}^{2} }}{{\lambda _{{leg}} L_{{leg}} w_{{leg}} t_{{leg}} }}\:{\text{and}}\:\beta = \frac{{h_{{rad}} }}{{\lambda _{{leg}} t_{{leg}} }} - TCR_{{Ni}} \alpha,$$

8 $$BC:\Delta T_{{leg}} (x = 0) = 0\:{\text{and}}\:\Delta T_{{leg}} (x = L_{{leg}} ) = \Delta T_{{mem}},$$

where $\lambda _{leg}$, is the equivalent thermal conductivity of the legs, $R_{leg}^{Ni}$ is the nickel electrical resistance in the leg, $TCR_{Ni}$ is the linearized TCR coefficient (around $0.47 \text{{K}}^{-1})$, $h_{rad}$ is the linearized radiation coefficient (in $\,\hbox {Wm}^{-2}$), $L_{leg}$, $w_{leg}$, $t_{leg}$ are respectively the length, the width and the thickness of the leg, and $\Delta T$ is the temperature rise of the membrane. Figure 5b depicts the analytical solution of Eq. (6) for the heated (current probes—red) and unheated (voltage probes—blue) legs of the far field membrane with a heating current $i_{in}=175 \mu A$, corresponding to a temperature rise of the membrane $\Delta T_{mem}=62 K$. We observe that the unheated leg profile is linear, showing that radiation has low influence on the temperature rise profile in the leg. This justifies the assumption that the radiation could be linearized. We obtained the thermal conductivity by fitting experimental data of heated 2-point probe leg resistance for the far field reference membrane Fig. 5c. We found $\lambda _{leg}^{eq}= 5.2 \, W m^{-1} K^{-1}$. As thermal radiation is low for this membrane, 10 times lower than Joule dissipated power in the legs, we have $\beta \approx \ -TCR_{Ni}\alpha$ in Eq. (7) and the temperature profile only depends on geometric parameters, dissipated power (experimentally estimated) and $\lambda$. However, the heated legs deviate from a linear profile showing the importance to consider Joule heating in the legs. After analytical resolution of the temperature profile in the legs, the conduction losses, $P_{cond} (\Delta T_{mem} )$, in the membrane is found:

9 $$\begin{aligned} P_{cond}(\Delta T_{mem})=2\lambda _{leg}w_{leg}t_{leg} \Bigg ( \Bigg [\frac{d\Delta T_{leg}(unheated)}{dx}\Bigg ]_{x=L_{leg}}+\Bigg [\frac{d\Delta T_{leg}(heated)}{dx}\Bigg ]_{x=L_{leg}}\Bigg ) \end{aligned}.$$

Deduced $P_{cond} (\Delta T_{mem} )$ is plotted on Fig. 2b. We observe that for a large temperature rise, conduction is lower for the membrane presenting high radiation losses (M2). For a given temperature rise, this membrane has a higher input current (as there is more radiation losses), so more heating power is dissipated in the legs compared to the far field reference. The result is that the conduction losses along the heated legs are lower for this membrane.

### Complex refractive index of $\hbox {SiO}_2$ and $\hbox {SiN}$ in mid infrared

Ellipsometry data of silicon oxide $\hbox {SiO}_2$ and silicon nitride $\hbox {SiN}$ were acquired with an IR-Vase ellipsometer with respectively $40^{\circ }$ and $65^{\circ }$ incident angle on thin blanket films directly deposited on silicon substrates with a thickness around ${200}\,\hbox {nm}$. The wavelength range was [${2}\,\upmu \hbox {m}$ ; ${17}\,\upmu \hbox {m}$]. Samples were pre-measured with a Woolam visible ellipsometer for which both films are completely transparent. We determined the exact thickness of the thin film *t* and the film high frequency permittivity $\varepsilon ^{inf}$, which facilitate IR ellipsometry data fitting. We fitted the IR ellipsometry data with Brendel oscillators^43^ (Fig. 5d $\Psi$ and $\Delta$ angles for $\hbox {SiO}_2$ (black) and $\hbox {SiN}$ (grey)):

10 $$\begin{aligned} \varepsilon (\nu )=\varepsilon ^{inf}+\sum _{i} \frac{1}{\sqrt{2\pi }\sigma _i}\int _{-\infty }^{\infty }exp \Bigg (\frac{(x-\nu _i^0)^2}{2\sigma _i^2)}\Bigg )\frac{{nu_i^p}^2}{x^2-\nu ^2-i\nu \nu _i^\tau }dx \end{aligned}$$

We fitted the ellipsometry data with one oscillator for $\hbox {SiN}$ and with two oscillators for $\hbox {SiO}_2$; oscillator values are resumed on the Table 1.

**Table 1:** Fitted Brendel oscillator parameters for $\hbox {SiN}$ and $\hbox {SiO}_2$.

$\hbox {SiN}$ $\varepsilon ^{inf}=3.8$ and $t=213 \hbox {nm}$					$\hbox {SiO}_2$ $\varepsilon ^{inf}=2.19$ and $t=215 \hbox {nm}$				
Oscillator	$\nu ^0$	$\nu ^\tau$	$\nu ^p$	$\sigma$	Oscillator	$\nu ^0$	$\nu ^\tau$	$\nu ^p$	$\sigma$
1	836.9	0.72	1106.7	144.0	1	1037.7	26.5	893.8	42.3
					2	1166.1	4.4	300.8	57.3

## Supplementary Information

Supplementary Information.
