# Supplementary material for: Toward applications of near-field radiative heat transfer with micro-hotplates
Source: Sci Rep. 2021 Jul 12;11:14347. doi: 10.1038/s41598-021-93695-7 (PMC8275596; doi:10.1038/s41598-021-93695-7)
Supplement: Supplementary file 1 — Supplementary Information. [file 41598_2021_93695_MOESM1_ESM.pdf]

# Supp. info : Toward Applications of Near-field Radiative Heat Transfer with Micro-hotplates

**Olivier Marconot[1,2,*], Alexandre Juneau-Fecteau[1,2], and Luc G. Fréchette[1,2]**

[1]Institut Interdisciplinaire d'Innovation Technologique (3IT), Université de Sherbrooke, Sherbrooke (QC),J1K 0A5, Canada
[2]Laboratoire Nanotechnologies Nanosystèmes (LN2) - CNRS UMI-3463, Université de Sherbrooke, Sherbrooke (QC),J1K 0A5, Canada
[*]olivier.marconot@usherbrooke.ca

## ABSTRACT

Bringing bodies close together at sub-micron distances can drastically enhance radiative heat transfer, leading to heat fluxes greater than the blackbody limit set by Stefan-Boltzmann law. This effect, known as near-field radiative heat transfer (NFRHT), has wide implications for thermal management in microsystems, as well as technological applications such as direct heat to electricity conversion in thermophotovoltaic cells. Here, we demonstrate NFRHT from microfabricated hotplates made by surface micromachining of $SiO_2$/SiN thin films deposited on a sacrificial amorphous Si layer. The sacrificial layer is dry etched to form wide membranes ($100\,\mu m$ x $100\,\mu m$) separated from the substrate by nanometric distances. Nickel traces allow both resistive heating and temperature measurement on the micro-hotplates. We report on two samples with measured gaps of $610\,nm$ and $280\,nm$. The membranes can be heated up to $250\,°C$ under vacuum with no mechanical damage. At $120\,°C$ we observed a 6.4 fold enhancement of radiative heat transfer compared to far-field emission for the smallest gap and a 3.5 fold enhancement for the larger gap. Furthermore, the measured transmitted power exhibits an exponential dependence with respect to gap size, a clear signature of NFRHT. Calculations of photon transmission probabilities indicate that the observed increase in heat transfer can be attributed to near-field coupling by surface phonon-polaritons supported by the $SiO_2$ films. The fabrication process presented here, relying solely on well-established surface micromachining technology, is a key step toward integration of NFRHT in industrial applications.

## Supplementary information contents:

- S1 : Fabrication process:
- S2 : Thickness influence on NFRHT:
- S3 : S3 : Calculation steps for Landauer-like formalism calculations:

## S1 : Fabrication process:

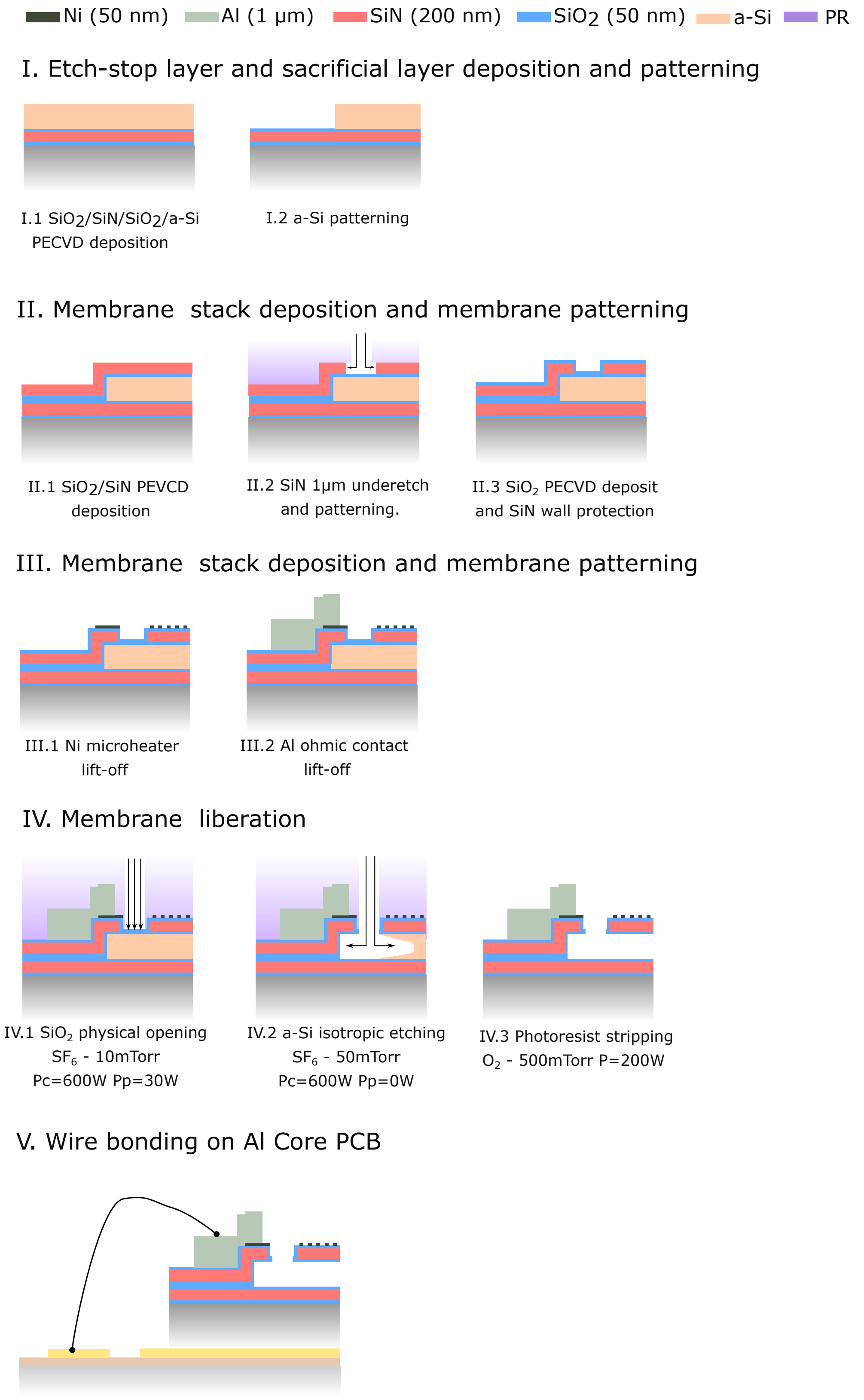


**Figure S1.** Fabrication process flow of NFRHT micro-hot plates

## S2 : Thickness influence on NFRHT and SPhP splitting:

NFRHT depends of the ONO stack total thickness. It drastically increases above 100 nm and saturates at 3 µm as shown on black curve on fig S2.a. However, it is also important to consider the conduction losses in legs. In our devices, the leg stack is the same than the membrane. By considering a simple conduction model, we plot on the right axis the ratio of radiation on conduction for a 100K membrane temperature elevation (grey curve on fig S2.a). We observe that the conduction/radiation ratio reach 0.6 for a total ONO thickness of 80 nm (instead of 300 nm in this work). We made a trade off to have a significant heat flux to measure ($1500 W/m^2$ for $\Delta T_{mem} = 100K$) and to keep a high ratio of radiation on conduction.

In our theoretical calculations, we attributed the two peaks on the spectral heat flux density near the SPhP to the splitting of short range (SR) SPhPs and long range (LR) SPhPs. We proved here this phenomenon by studying a simple configuration of thin $SiO_2$ membrane above a silicon substrate. Fig. S2.b plots the spectral heat flux density for evanescent waves in TM polarization, where SPhPs play an important role. We clearly see a splitting of the peak as the thickness decreases. This behaviour is characteristic of long range and short range SPhP splitting.

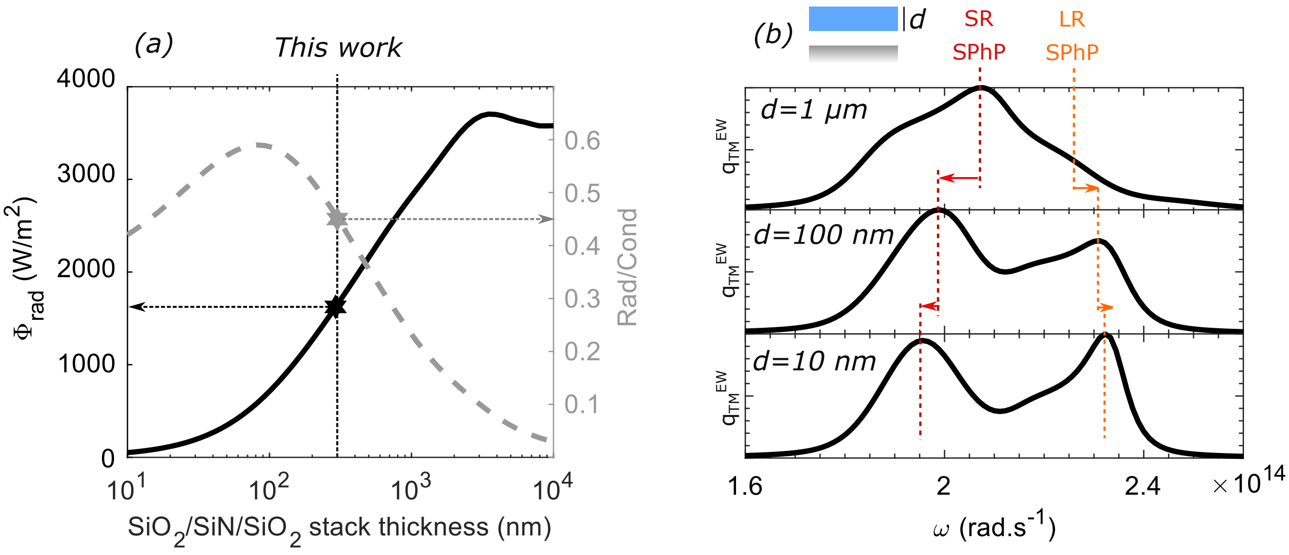


**Figure S2.** (a) influence of $SiO_2/SiN/SiO_2$ total thickness stack on radiative heat transfer (black line) and ratio on thermal losses in legs for $\Delta T_{mem} = 100K$ (b) Spectral heat flux density for evanescent waves in TM polarisation for a simple three body configuration {black body env ; $SiO_2$ membrane ; Si substrate} - evidence of splitting of long range (LR) and short range (SR) SPhP while $SiO_2$ thickness decreases

## S3 : Calculation steps for Landauer-like formalism calculations

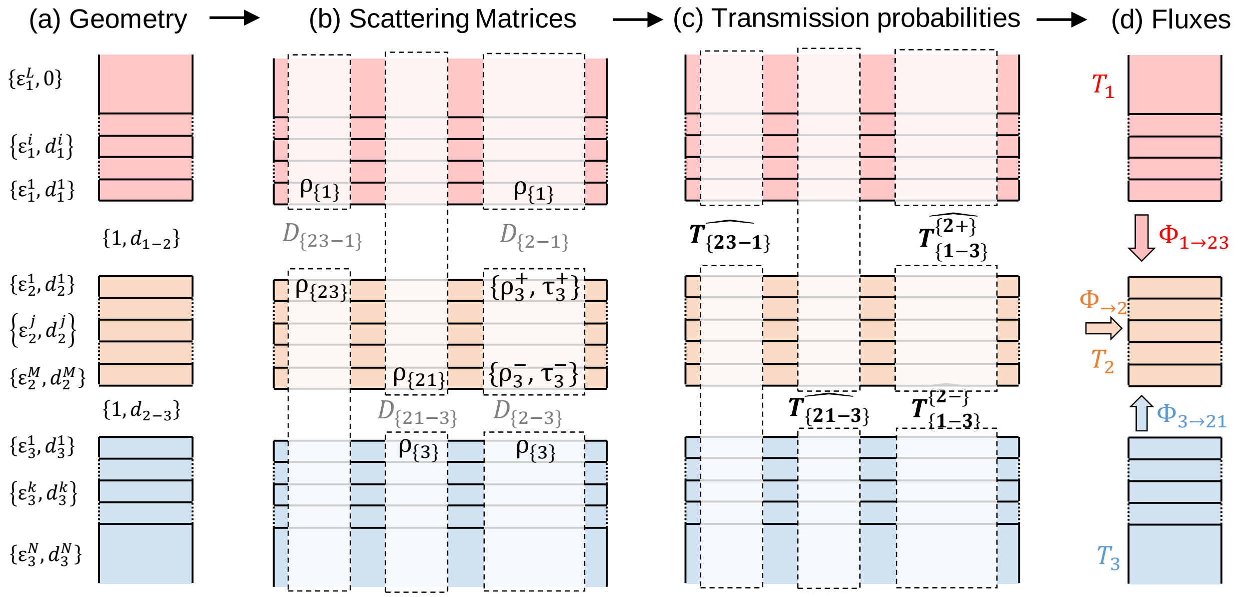


**Figure S3.** Calculation steps for transmission probability matrices

1. Discretization of the $(\omega, k_{||})$ space.
2. Definition of the three body structure as shown on Fig.S3.a with :
   - the $(\varepsilon_i(\omega), d_i)$ definition for each layer in each body.
   - the vacuum gap between each body $(d_{3-2}, d_{1-2})$
   - The temperature of each body $(T_1, T_2, T_2)$
3. Complex Fresnel transmission and reflection coefficient at each interface $(i, j)$ for both polarization:
   - Calculation of the wave vector component parallel to the surface for each layer :

$$k_\perp^i = \sqrt{\varepsilon_i^2 \frac{\omega^2}{c^2} - k_{||}^2}$$

   - Calculation of Fresnel reflection coefficient in both polarization :

$$r_{i,j}^{TE} = \frac{k_\perp^i - k_\perp^j}{k_\perp^i + k_\perp^j} \qquad r_{i,j}^{TM} = \frac{\varepsilon^j k_\perp^i - \varepsilon^i k_\perp^j}{\varepsilon^j k_\perp^i + \varepsilon^i k_\perp^j}$$

   - Fresnel transmission coefficient in both polarization :

$$t_{i,j}^{TE} = \frac{2k_\perp^i}{k_\perp^i + k_\perp^j} \qquad t_{i,j}^{TM} = \frac{2k_\perp^i \sqrt{\varepsilon^j \varepsilon^i}}{\varepsilon^j k_\perp^i + \varepsilon^i k_\perp^j}$$

4. Complex reflection and transmission coefficients for each body (1;2;3) using scattering matrix formalism and the two body assembly (12;32) for both polarization:
   - Scattering matrix initialisation:

$$\mathbf{S} = \mathbf{I_2}$$

- Iterative calculation (with $\delta_i = k_\perp^i d_i$):

$$\mathbf{S_{11}}(0,i+1) = \frac{\mathbf{S_{11}}(0,i)t_{i,i+1}e^{i\delta_i}}{1-\mathbf{S_{11}}(0,i)r_{i,i+1}e^{2i\delta_i}}$$
$$\mathbf{S_{12}}(0,i+1) = \frac{\mathbf{S_{12}}(0,i)e^{2i\delta_i}-r_{i,i+1}}{1-\mathbf{S_{12}}(0,i)r_{i,i+1}e^{2i\delta_i}}$$
$$\mathbf{S_{21}}(0,i+1) = \frac{\mathbf{S_{11}}(0,i+1)\mathbf{S_{22}}(0,i)r_{i,i+1}e^{i\delta_i}}{t_{i,i+1}} + \mathbf{S_{21}}(0,i)$$
$$\mathbf{S_{22}}(0,i+1) = \frac{\mathbf{S_{22}}(0,i)[r_{i,i+1}\mathbf{S_{12}}(0,i+1)+1]e^{i\delta_i}}{t_{i,i+1}}$$

- Calculation of the total reflection and complex coefficients for each body (or assembly) :

$$\rho^+ = \mathbf{S_{21}}(0,N+1) \quad \rho^- = \mathbf{S_{12}}(0,N+1)$$
$$\tau^+ = \mathbf{S_{11}}(0,N+1) \quad \tau^- = \mathbf{S_{22}}(0,N+1)$$

5. Fabry-Perot coefficients between each body and body assembly:

$$D_{i2-j} = 1-\rho_{i2}\rho_j e^{2i\delta_{i2-j}} \text{ with } \delta_{i2-j} = k_\perp d_{2-j}$$
$$D_{2-j} = 1-\rho_{2}\rho_j e^{2i\delta_{2-j}} \text{ with } \delta_{2-j} = k_\perp d_{2-j}$$

6. Transmission probability matrices (with $i=\{1,3\}$, $j=\{1,3\}_{i\neq j}$, $\pm = \{+ \text{ if } i=3, - \text{ if } i=1\}$):

- Transmission probability from two-body assembly $\{i2\}$ to one body $\{j\}$ :

$$\hat{\mathbf{T}}_{\mathbf{i2-j}} = \frac{(1-|\rho_{i2}|^2)(1-|\rho_j|^2)}{|D_{i2-j}|^2} \text{ for propagating waves } (k_{||} < \omega/c)$$
$$\hat{\mathbf{T}}_{\mathbf{i2-j}} = \frac{4Im(\rho_{i2})Im(\rho_j)e^{2ik_{||}d_{2-j}}}{|D_{i2-j}|^2} \text{ for evanescent waves } (k_{||} > \omega/c)$$

- Transmission probability from body $\{i\}$ to body $\{j\}$ *through* body $\{2\}$ :

$$\hat{\mathbf{T}}^{\mathbf{2}\pm}_{\mathbf{i-j}} = \frac{|\tau_2^{\pm}|^2(1-|\rho_i|^2)(1-|\rho_j|^2)}{|D_{i2-j}|^2|D_{2-j}|^2} \text{ for propagating waves } (k_{||} < \omega/c)$$
$$\hat{\mathbf{T}}^{\mathbf{2}\pm}_{\mathbf{i-j}} = \frac{4|\tau_2^{\pm}|^2 Im(\rho_i)Im(\rho_j)e^{2ik_{||}d_{i-j}}}{|D_{i2-j}|^2|D_{2-j}|^2} \text{ for evanescent waves } (k_{||} > \omega/c)$$

7. Spectral heat flux density calculations:

- Difference of thermal distribution definition between two body:

$$\Delta n_{ij}(\omega) = \frac{1}{e^{\hbar\omega/k_b T_i}-1} - \frac{1}{e^{\hbar\omega/k_b T_j}-1}$$

- Heat flux spectral density from $i=\{1,3\}$ to $\{2\}$ (with $j=\{1,3\}_{i\neq j}$)

$$q_{i\to 2j}(\omega) = \frac{\hbar\omega}{4\Pi^2}\sum_{pol}\int_k k_{||}[\hat{\mathbf{T}}_{\mathbf{j2-i}}\Delta n_{i2} - \hat{\mathbf{T}}^{\mathbf{2}\pm}_{\mathbf{j-i}}\Delta n_{j2}]dk_{||}$$

8. Energy balance on body $\{2\}$

- Integration over $\omega$ to obtain the net radiative heat flux:

$$\Phi_{i\to 2j} = \int_\omega q_{i\to 2j} d\omega$$

- Energy balance on the second body:

$$\Phi_{\to 2} = \Phi_{3\to 21} + \Phi_{1\to 23}$$
